# The Book World of Medicine and Science

**Published:** 1901-04-27

**Authors:** 


### The Book World of Medicine and Science.

**Laboratory Directions for Beginners in Bacteriology.** By Veranus A. Moore, B.S., M.D. 2nd Edition revised and enlarged. (Boston, U.S.A.: Geen & Co. Pp. 143. Illustrated. 1900.)

This is a very excellent little manual. It does not profess to teach bacteriology or to be even a compendium of technique. But it provides the student who is just beginning the study of bacteriology with an outline of the work that teachers of experience think it best for him to undertake. Particular things are chosen for him to do, and particular methods are chosen for him to employ, and the student who conscientiously follows the directions given will gain a working knowledge of most essential methods and a definite knowledge of many important species of bacteria. The plan of the book is very simple. At the commencement a tabular statement of those journals, periodicals and text-books of value to the student is furnished, and convenient lists are given: (*a*) of laboratory apparatus for general use; (*b*) of apparatus for individual use; (*c*) of material to be provided by the student. Twelve pithy laboratory maxims preface the subject-matter proper of the book, which is divided into 64 exercises or lessons. These exercises comprise the cleaning of glass ware, the preparation of tubes, dishes, and nutrient media and other practices necessary to the student, but almost invariably omitted from what is familiar to London students as "post-graduate bacteriology"—a science taught in half-a-dozen "demonstrations." The student is led by Dr. Moore to proceed from these uninteresting, but most important, exercises to the examination of cultures and colonies, to the identification and study of typical organisms, to the testing of water and of disinfectants, and to the isolation of organisms from diseased tissues and morbid fluids. An appendix deals with certain supplementary details and there is an adequate index. Dr. Moore's little book is one of the best laboratory handbooks that we are acquainted with and reflects credit on American teachers. In its lucidity and in the mastery of craft that it displays there is something Celtic rather than Teutonic, and its thoroughness puts to shame much of the superficial speculation and careless technique that is becoming characteristic of English bacteriology. We trust that Dr. Moore's very admirable little manual will speedily find its way into many an English laboratory.

**On Sanitary and other Matters.** By George S. Keith, M.D., LL.D. (London: Adam and Charles Black. 1900. Pp. 126. Price 2s. 6d.)

A few years ago considerable attention was paid in the lay press, to a work entitled "A Plea for a Simpler Life." Dr. George Keith, who wrote that book, is the author of the collection of essays now before us. But, as Dr. Keith himself naively observes in the preface, the essays are of "a somewhat mixed character." Further, although the author believes some of them to be "better fitted for the pages of a daily or weekly paper," he confesses that with but one exception they have been declined by those editors to whom he has submitted them. It is a little difficult to treat Dr. Keith's essays seriously. He is concerned at the "waste of water" permitted to dwellers in towns, and suggests that Englishmen should, from an economic motive, abandon their daily bath and content themselves instead with a rub down by a "towel lightly wrung out of tepid water." We commend Dr. Keith and his advocacy of what Sam Weller called a "dry rinse" to the secretaries of monopolist water companies. It seems only congruous that we should pass from Chapter I.—a crusade against the bath—to Chapter II., in which we learn that the main object of a sanitary authority or physician must be, not to stamp out scarlet fever, but to arrange in what manner "the disease may give least trouble and expense to the public." Dr. Keith therefore advises that when scarlet fever "gets into a family, every facility should be given to the spread of the disease over all liable to it." Dr. Keith says that he has followed this plan for fifteen years, and has never had occasion to regret having done so. We trust that his patients are equally free from regret. The remainder of the book is largely devoted to discursive reminiscences which form an illogical basis for the recommendation of practices called by Dr. Keith "self massage." What self massage exactly is does not quite appear. But the omission of details in the text has evidently not escaped notice, for we have found between the title page and fly-leaf a printed slip bearing the name and address of Mr. A. Keith, "Masseur and Teacher of Self Massage."

**First Aid to the Injured, with Special References to Accidents Occurring in the Mountains.** By Dr. Oscar Bernhard. Translated from the German by Michael Foster, M.D. (London: T. Fisher Unwin. 1900. Illustrated. Price 2s. 6d.)

Dr. Bernhard, the well-known surgeon to the Engadine Hospital, has produced in this little book a work deserving the highest commendation. Books innumerable on ambulance work have appeared in the last few years, but there is not one which we can so heartily commend to the Alpine climber as this.

It is not an amateur production, but is written by an experienced surgeon who is also an expert mountaineer, and therefore is familiar with the accidents that occur in climbing, and is, moreover, a master of surgical improvisation. The lithographed illustrations are amongst the most appropriate, and at the same time artistic ones, that we have ever seen in a work of this nature; and the mountaineer, with this little book in his knapsack, will be able at once, by its help, to arrange for the transport of an injured companion under the most adverse circumstances. Finally, we must not omit reference to the excellent manner in which Dr. Foster, of San Remo, has performed the task of translation. The printers and bookbinders, too, have paid attention to every little detail that can make the book convenient for a traveller's pocket or knapsack.

